# The Origin of COVID-19 and Why It Matters

**DOI:** 10.4269/ajtmh.20-0849

**Published:** 2020-07-22

**Authors:** David M. Morens, Joel G. Breman, Charles H. Calisher, Peter C. Doherty, Beatrice H. Hahn, Gerald T. Keusch, Laura D. Kramer, James W. LeDuc, Thomas P. Monath, Jeffery K. Taubenberger

**Affiliations:** 1 American Committee on Arthropod-Borne Viruses, American Society of Tropical Medicine and Hygiene, Arlington, Virginia;; 2 National Institute of Allergy and Infectious Diseases, National Institutes of Health, Bethesda, Maryland;; 3 American Society of Tropical Medicine and Hygiene, Arlington, Virginia;; 4 Arthropod-borne and Infectious Diseases Laboratory, Department of Microbiology, Immunology & Pathology, College of Veterinary Medicine and Biomedical Sciences, Colorado State University, Fort Collins, Colorado;; 5 Department of Microbiology and Immunology, University of Melbourne at the Doherty Institute, Melbourne, Australia;; 6 Department of Medicine, Perelman School of Medicine, University of Pennsylvania, Philadelphia, Pennsylvania;; 7 Department of Microbiology, Perelman School of Medicine, University of Pennsylvania, Philadelphia, Pennsylvania;; 8 Department of Medicine, Boston University School of Medicine, Boston, Massachusetts;; 9 Department of Global Health, Boston University School of Public Health, Boston, Massachusetts;; 10 National Emerging Infectious Diseases Laboratory at Boston University, Boston, Massachusetts;; 11 Arbovirus Laboratory, Wadsworth Center, New York State Department of Health, Albany, New York;; 12 Department of Biomedical Sciences, School of Public Health, State University of New York at Albany, Albany, New York;; 13 Galveston National Laboratory and Department of Microbiology and Immunology, University of Texas Medical Branch, Galveston, Texas;; 14 Crozet BioPharma LLC, Devens, Massachusetts;; 15 Viral Pathogenesis and Evolution Section, Laboratory of Infectious Diseases, National Institute of Allergy and Infectious Diseases, National Institutes of Health, Bethesda, Maryland

## Abstract

The COVID-19 pandemic is among the deadliest infectious diseases to have emerged in recent history. As with all past pandemics, the specific mechanism of its emergence in humans remains unknown. Nevertheless, a large body of virologic, epidemiologic, veterinary, and ecologic data establishes that the new virus, SARS-CoV-2, evolved directly or indirectly from a β-coronavirus in the sarbecovirus (SARS-like virus) group that naturally infect bats and pangolins in Asia and Southeast Asia. Scientists have warned for decades that such sarbecoviruses are poised to emerge again and again, identified risk factors, and argued for enhanced pandemic prevention and control efforts. Unfortunately, few such preventive actions were taken resulting in the latest coronavirus emergence detected in late 2019 which quickly spread pandemically. The risk of similar coronavirus outbreaks in the future remains high. In addition to controlling the COVID-19 pandemic, we must undertake vigorous scientific, public health, and societal actions, including significantly increased funding for basic and applied research addressing disease emergence, to prevent this tragic history from repeating itself.

In 2007, scientists studying coronaviruses warned: “The presence of a large reservoir of SARS-CoV–like viruses in horseshoe bats… is a time bomb. The possibility of the re-emergence of SARS and other novel viruses… should not be ignored.”^1^

Few paid attention following the disappearance of SARS after the initial outbreak in 2002. Now, 18 years later, COVID-19 has emerged as the deadliest respiratory disease pandemic since 1918, when the “Spanish” influenza pandemic killed an estimated 50 million people.^2^ We need to understand what happened so that we can prevent it from happening again, and be better prepared to contain similar pandemics at their outsets.

## EMERGENCE OF THE COVID-19 PANDEMIC

The agent of COVID-19, SARS-CoV-2, was named after the genetically related SARS-CoV (more recently distinguished by some as SARS-CoV-1), which caused a deadly near-pandemic in 2002–2003.^3^ Before 2019, neither SARS-CoV-2 nor its genetic sequences had ever been identified in viruses of humans or animals.

Even so, scientific research conducted over the last two decades provides clues about how and why the COVID-19 pandemic appeared. We must understand these critically important scientific findings, described in the following text, so that we can better address significant existential risks we will continue to face for the foreseeable future.

## HOW VIRAL DISEASES EMERGE

Viruses are compact nucleic acid packages of either DNA or (in the case of coronaviruses) RNA associated with proteins, and in some cases with lipids. Viruses are not living organisms and can only reproduce inside living cells susceptible to viral entry and with the capacity to replicate viral nucleic acids and translate nucleic acid signals into amino acids to build viral proteins. Viruses are therefore nonliving self-contained genetic programs capable of redirecting a cell’s machinery to produce more of themselves.

It follows that when a virus enters a human cell for the first time, it has very recently been transmitted from cells of some other host, that is, from another animal or, for example, an insect vector. Emergence of a pathogen between a vertebrate or an insect has been referred to as host-switching, sometimes described as a spillover event. Most of the human viral and nonviral infectious diseases that have existed for centuries—measles, influenza, cholera, smallpox (eradicated in 1980), falciparum malaria,^4^ dengue, HIV, and many others—originated by animal-to-human host-switching.^5^ The complex genetic events that underlie host-switching differ greatly from pathogen to pathogen, but general mechanisms have been recognized for many.^6–9^

Host-switching determinants prominently include social, environmental, and biological factors providing the opportunity for host–species interaction; shared host cell receptors; genetic distance between transmitting and receiving hosts; and characteristics and complexity of the viral quasi-species or viral swarm. (RNA viruses in particular are not transmitted to multiple cells as identical virions, but as collections of thousands of different genetically related virions. The ever-changing complexity of the viral swarm varies among species, genetically distinct but related individuals of the same species, and in single hosts over time.)

Studying animal viruses that have previously spilled over into humans provides clues about host-switching determinants. A well-understood example is influenza virus emergence into humans and other mammals.^2^ Human pandemic and seasonal influenza viruses arise from enzootic viruses of wild waterfowl and shore birds. From within this natural reservoir, the 1918 pandemic “founder” virus somehow host-switched into humans. We know this from genetic studies comparing avian viruses, the 1918 virus, and its descendants, which have caused three subsequent pandemics, as well as annual seasonal influenza in each of the 102 years since 1918. Similarly, other avian influenza viruses have host-switched into horses, dogs, pigs, seals, and other vertebrates, with as yet unknown pandemic potential.^2,10,11^ Although some molecular host-switching events remain unobserved, phylogenetic analyses of influenza viruses allow us to readily characterize evolution and host-switching as it occurs in nature.^2^

## CORONAVIRUSES

**Figure 1.**
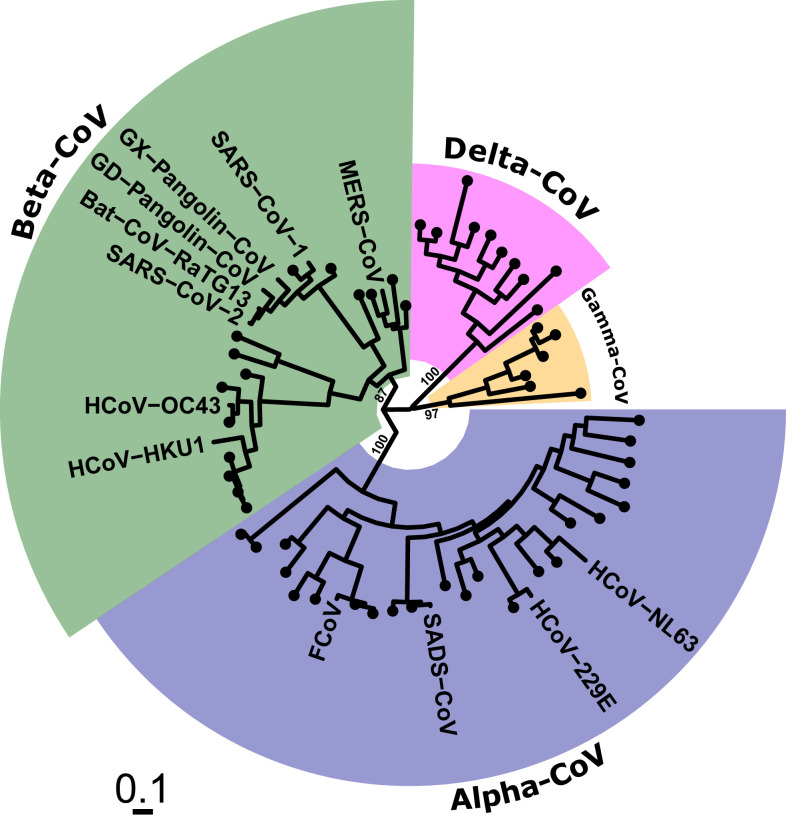
Phylogenetic relationships of selected coronaviruses of medical and veterinary importance. Human SARS-CoV and SARS-CoV-2 are closely related to numerous bat and pangolin coronaviruses in a viral genetic grouping called sarbecoviruses, which contains many other viruses very closely related to SARS-CoV and SARS-CoV-2. These viruses belong to the order *Nidovirales*, family *Coronaviridae*, subfamily *Coronavirinae* and the four genera *Alphacoronavirus*, *Betacoronavirus*, *Gammacoronavirus*, and *Deltacoronavirus*. The betacoronaviruses are comprised of two subgenera, *Sarbecovirus* and *Merbecovirus*. The former include SARS-CoV and SARS-CoV-2; the latter includes Middle East respiratory syndrome-related coronavirus (MERS-CoV). Image created by Sebastian M. Gygli, Ph.D., NIAID, NIH, and used with permission.

Coronaviruses are RNA viruses globally distributed in a large but unknown number of animal species. Coronaviruses important for humans are found within phylogenetically distinct taxonomic subgroups, labeled as the α- and β-coronaviruses (Figure 1).^12^ Four endemic human coronaviruses, which emerged at some undetermined time in the past, cause (mostly) mild self-limited upper respiratory tract infections (Figure 1).

## RECENT CORONAVIRUS EMERGENCES FROM ANIMALS INTO HUMANS

Until recently, relatively little was known about coronaviruses, and research interest in these common cold viruses was minimal. Eighteen years ago, a previously unknown β-coronavirus named SARS-CoV suddenly emerged. Following its initial appearance in China it spread to 29 other countries, causing a near-pandemic and killing 813 of the 8,809 people with confirmed infection before being controlled by aggressive public health measures. It has not been seen since. In 2012, however, another previously unknown β-coronavirus named Middle East respiratory syndrome coronavirus (MERS-CoV), and closely related to SARS-CoV, emerged to cause high case-fatality human infections. Fortunately, this virus does not efficiently transmit between humans, and cases have been largely limited to the Middle East where its intermediary host, the dromedary camel, is present in relatively high numbers. In 2016, yet another novel bat-origin coronavirus, an α-coronavirus, emerged in China to cause a novel epizootic disease in pigs, termed swine acute diarrhea syndrome coronavirus (SADS-CoV). And most recently, at least as early as late November 2019, SARS-CoV-2 was recognized and became the third fatal bat virus–associated human disease emergence and the fourth bat virus–associated mammalian emergence in 18 years.

## CORONAVIRUS EMERGENCE RISKS

An enormous reservoir of coronaviruses infects hundreds of bat species distributed globally. SARS-CoV, MERS-CoV, and SARS-CoV-2 are closely related β-coronaviruses clustering in two adjacent phylogenetic groupings: sarbecovirus (SARS-like viruses) and merbecovirus (MERS-like viruses) (Figure 1). The two SARS viruses, as well as SADS-CoV, are descended from viruses enzootic in rhinolophid (genus, *Rhinolophus*), or horseshoe bats.

Over the past 15 years, scientists have also identified global animal reservoirs of coronaviruses (in Africa, the Americas, the Middle East, Asia and Southeast Asia, and particularly China, the location of three of the four most recent emergences). These efforts have revealed much about coronaviral ecosystems, reservoir hosts, viral movement between hosts, viral evolution, and risk of emergence into humans and other mammals.

Bats of numerous globally distributed genera and species are now known to be the major reservoir of animal coronaviruses. One 20-country study of more than 19,000 animals (predominantly nonhuman primates, bats, and rodents) revealed that bats accounted for more than 98% of coronavirus detections, and that almost 9% of > 12,000 randomly studied bats were infected with one or more coronavirus.^13^ Significant interspecies viral transmission between closely and distantly related bats also appears to be important. Bats of some species, including rhinolophids, co-roost with bats of other species, facilitating viral exchanges and enhanced viral evolution associated with genetic recombination. In fact, many such bat coronaviruses have genetic sequences similar to SARS-CoV and SARS-CoV-2.

**Figure 2.**
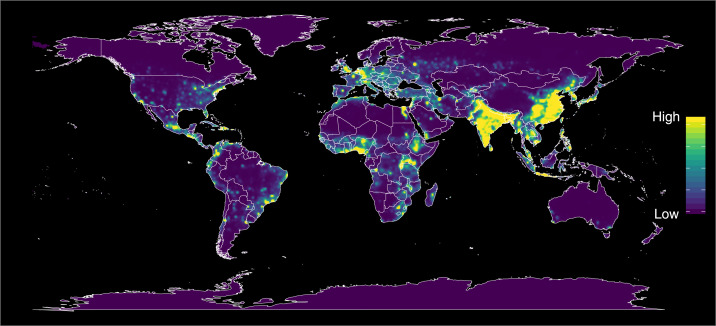
Predicted global hotspots for disease emergence, showing estimated risks, adjusted for reporting bias. From a comprehensive global study combining multiple data sources. Reproduced with permission from Allen et al*.*^14^

Investigators have also mapped global hotspots for potential infection emergence, prominently in south/southwest China and contiguous regions and countries (Figure 2),^14^ and have identified numerous human–animal interactions that constitute emergence risk factors, for example bat tourism, wet markets, wildlife supply chains for human consumption,^15^ land management practices, and environmental perturbations.^16–18^ Virologic and risk mapping studies indicate a very high risk of further coronavirus outbreaks.^19–21^

SARS-CoV and SARS-CoV-2 emerged in China, home to bats of more than 100 species, many of which carry α- and/or β-coronaviruses. In one study, more than 780 partial coronavirus genetic sequences were identified from bats of 41 species infected by α- and of 31 species infected by β-coronaviruses.^21^ Within the sarbecovirus lineage, encompassing SARS and SARS-like viruses, many identified genetic sequences are very similar to SARS-CoV and SARS-CoV-2.^21–23^ One such virus is more than 96% identical to SARS-CoV-2 in its whole genome^23^; another shares more than 97% identity in the 1ab replicase gene, as well as a furin cleavage site insertion.^24^ Nature is clearly a cauldron for intense and dangerous coronavirus evolution.

## WAS COVID-19 PREDICTED?

A clearer, more worrisome picture of the coronavirus ecosystem has recently come together. A contiguous area encompassing parts of south/southwest China, Laos, Myanmar, and Vietnam constitutes a bat coronavirus “hotspot,” featuring intense interspecies viral transmission. In such hotspots, a rich diversity of SARS-like viruses has been found, not only in rhinolophid bats but also in bats of other genera and species to which these viruses had host-switched. The same rhinolophid bats are also implicated in the emergence of SADS-CoV in southern China. Many of these SARS-like viruses bind to human angiotensin-converting enzyme-2 (ACE2) receptors and infect human respiratory epithelial cells in vitro, suggesting their pandemic potential.^19,25^

Ominously, bat-to-human transmission of SARS-like viruses has already been detected,^20^ perhaps representing pandemic near-misses. Even the more genetically distant SADS-CoV infects cells of humans and numerous other vertebrates, raising concern about indirect coronavirus emergences. This seems to have occurred with the bat-to-camel-to-human emergence of MERS, and possibly with SARS-CoV emergence into humans, which may have resulted from bat virus infection of masked palm civet cats (*Paguma larvata*), with subsequent human spillover.^12^ As a byproduct of the important international surveillance work described above, in 2017, the therapeutic benefit of the antiviral drug remdesivir was suggested; it is now, in 2020, being widely used to treat persons infected with SARS-CoV-2.^26^

Since 2007, when alarming predictions about threatened coronavirus emergences began to appear,^1^ understanding of coronavirus ecosystems has become far more complete. Over the past 5 years, Chinese, American, European, and other scientists have begun to renew warnings that humans are intensively interacting with coronavirus-infected bats, that enzootic SARS-related bat coronaviruses have all of the essential components of the SARS virus, that some of these SARS-like viruses can infect laboratory-humanized mice to cause SARS-like disease, that SARS-like viruses have the ability to directly infect and be transmitted between humans, and, therefore, that these viruses are poised for human emergence.^19,21,22^ Many scientists have proposed aggressive monitoring of known hotspots to try to predict and prevent viral emergence that might impact human health, including early warning of host-switching events.^19,20,27^

Unfortunately, outside of some members of the scientific community, there has been little interest and no sense of urgency. In 2020, we learned, tragically, what 12 years of unheeded warnings have led to: a bat-derived sarbecovirus—from the very same SARS-like bat virus group that had been warned about by multiple voices for over a decade—emerged and proceeded to cause the COVID-19 pandemic that now sweeps the globe.

SARS-CoV-2 emerged essentially as predicted: a natural event associated with either direct transmission of a bat coronavirus to humans or indirect transmission to humans via an intermediate host such as a Malaysian pangolin (*Manis javanica*) or another, yet-to-be-identified mammal.^28–31^

It should be clarified that theories about a hypothetical man-made origin of SARS-CoV-2 have been thoroughly discredited by multiple coronavirus experts.^21,28,29^ SARS-CoV-2 contains neither the genetic fingerprints of any of the reverse genetics systems that have been used to engineer coronaviruses nor does it contain genetic sequences that would have been “forward engineered” from preexisting viruses, including the genetically closest sarbecoviruses. That is, SARS-CoV-2 is unlike any previously identified coronavirus from which it could have been engineered. Moreover, the SARS-CoV-2 receptor-binding domain, which has affinity for cells of various mammals, binds to human ACE2 receptors via a novel mechanism.

Engineering such a virus would have required 1) published or otherwise available scientific knowledge that did not exist until after COVID-19 recognition; 2) a failure to follow obvious engineering pathways, resulting in an imperfectly constructed virus; and 3) an ability to genetically engineer a new virus without leaving fingerprints of the engineering. Furthermore, the 12 amino acid furin-cleavage site insertion between the SARS-CoV-2 spike protein’s S1 and S2 domains, which some have alleged to be a sign of genetic engineering, is found in other bat and human coronaviruses in nature, probably arising via naturally occurring recombination.^24^

It is also highly unlikely that SARS-CoV-2 was released from a laboratory by accident because no laboratory had the virus nor did its genetic sequence exist in any sequence database before its initial GenBank deposition (early January 2020). China’s laboratory safety practices, policies, training, and engineering are equivalent to those of the United States and other developed countries,^32^ making viral “escape” extremely unlikely, and of course impossible without a viral isolate present. SARS-CoV-2 shares genetic properties with many other sarbecoviruses, lies fully within their genetic cluster, and is thus a virus that emerged naturally.

## COVID-19 EMERGENCE MECHANISMS: WHY THEY MATTER

Understanding how COVID-19 emerged is of great importance. We now know that the viruses causing SARS, MERS, and COVID-19 are all members of enormous groups of bat coronaviruses distributed globally, and that many of these viruses are functionally preadapted to human emergence. This preadaptation can be thought of as “accidental” because it must have occurred in nature in the absence of human infection and does not rule out further human adaptation to enable pandemicity. Molecular mechanisms of preadaptation are not fully known, but are undoubtedly related to functional similarities between ACE2 receptors on the cells of numerous mammals (bats, humans, minks, cats, and other domestic and wild animals).^33,34^

The ability of coronaviruses to evolve at a high rate, illustrated by extreme phylogenetic diversity, coupled with the dispersion of new viral variants within an enormous array of wild animal species that can serve as hosts, portends poorly for the future of coronavirus disease emergence. We are already seeing coronavirus mutants with altered affinity for human ACE2. Whether bat coronaviruses evolve independently or by “sampling” various mammalian ACE2 receptors, the result is the same. That bat sarbecoviruses so easily switch between multiple hosts suggests a many-pronged human risk: directly from bats and indirectly from other mammals infected by bat viruses. Because we have only just begun to sample, sequence, and study bat/mammalian coronaviruses, we can be certain that what we now know is but the tip of a very large iceberg.

The findings described earlier reaffirm what has long been obvious: that future coronavirus transmissions into humans are not only possible, but likely. Scientists knew this years ago and raised appropriate alarm. Our prolonged deafness now exacts a tragic price.

The story of COVID-19 emergence sends a powerful message. A quantum leap in bat coronavirus surveillance and research is urgently needed. This work must emphasize virologic and behavioral field studies of humans and animals wherever they interface, and especially in disease hotspots, as well as virologic studies related to human and animal spillover risks and the means of reducing them.^35^

Important research that has languished, been underfunded, or discontinued should be greatly expanded to deal with the urgency of the situation, and more scientists, including scientists working in China and other hotspot countries (Figure 2), should be recruited to these efforts, especially in international research partnerships. Full, open international collaboration involving many countries is essential. In particular, field research on the prevalence and virus-host relationships of coronaviruses, development of platform technologies for diagnostics, vaccines, and animal models for studies of pathogenesis and potential therapeutics is essential to permit, for example, modeling structure/function relationships of specific binding domains from newly identified agents to create critical tools for disease control.

In addition to robust expansion of surveillance and research, there are things that we can do now to lower our risks. We know much about coronavirus hotspots, not only in China but also globally; we can more aggressively surveil these locations to learn more about the local viral ecology and identify initial human spillover events. We also know much about human behaviors that directly and indirectly bring us into contact with bats, including risks from wet markets, bat cave tourism, capturing and eating bats, and perturbing the environment in ways that alter bat habitats and habits. These are behaviors that we can and must change.

We can also strengthen basic public health, including hygiene and sanitation, so that emerging viruses do not have a fertile field in which to amplify replication, and we must build and maintain strong public health infrastructure to respond quickly and efficiently to pathogen emergence. For viruses that have emerged, such as SARS-CoV-2, we need to develop effective antivirals and, ideally, broadly protective vaccines. Education and communication with populations where spillover events occur is also an important component of risk reduction.

We must also realize that the problem is larger than just coronaviruses. In recent years, we have seen emergences and reemergences of numerous other human infectious diseases such as Ebola fever, Lassa fever, hantavirus pulmonary syndrome, human monkeypox, HIV, dengue, chikungunya, Zika, and epizootic avian influenza. We have entered a new pandemic era,^36^ one in which epidemic and pandemic emergences are becoming commonplace; some are likely to be highly pathogenic. In 2020, our science is sufficiently robust to have a good chance of controlling pandemic viral emergences within 2–3 years, but dramatically insufficient to prevent and control their emergences in the first place.

We should begin developing broadly protective vaccines and broadly therapeutic antiviral/antimicrobial agents against pathogens within taxonomic groups likely to emerge in the future, including coronaviruses, henipaviruses, and filoviruses, among others. Organizations like the Coalition for Epidemic Preparedness Innovations, among others, should be extended and strengthened, emphasizing, in addition to vaccine development, therapeutics as well as prevention tools. Pandemic prevention should be a global effort on a par with chemical and nuclear weapon prevention.

Unless we reset the equation; invest more in critical and creative laboratory, field, and behavioral research; and start finding ways to prevent these emergences, we will soon see additional coronavirus pandemics, as well as global spread of other types of infectious agents not yet imagined, caused by some of the millions of viruses in the natural world, many of which we have not yet had the time and funding to identify and study.^27^

Understanding how COVID-19 emerged is a critical point on a steep learning curve we must quickly master. As we face the mounting deaths and societal upheavals of the COVID-19 pandemic, we must not lose sight of how this pandemic began, how and why we missed the warning signs, and what we can do to prevent it from happening again—and again.
